# Metataxonomic profiling and prediction of functional behaviour of wheat straw degrading microbial consortia

**DOI:** 10.1186/1754-6834-7-92

**Published:** 2014-06-12

**Authors:** Diego Javier Jiménez, Francisco Dini-Andreote, Jan Dirk van Elsas

**Affiliations:** 1 Department of Microbial Ecology, Center for Ecological and Evolutionary Studies (CEES), University of Groningen (RUG), Nijenborgh 7, 9747AG Groningen, The Netherlands

**Keywords:** Soil, Lignocellulose, Wheat straw, Microbial consortia, PICRUSt, *Klebsiella*, *Trichosporon*, alpha-L-fucosidases, beta-xylosidases

## Abstract

**Background:**

Mixed microbial cultures, in which bacteria and fungi interact, have been proposed as an efficient way to deconstruct plant waste. The characterization of specific microbial consortia could be the starting point for novel biotechnological applications related to the efficient conversion of lignocellulose to cello-oligosaccharides, plastics and/or biofuels. Here, the diversity, composition and predicted functional profiles of novel bacterial-fungal consortia are reported, on the basis of replicated aerobic wheat straw enrichment cultures.

**Results:**

In order to set up biodegradative microcosms, microbial communities were retrieved from a forest soil and introduced into a mineral salt medium containing 1% of (un)treated wheat straw. Following each incubation step, sequential transfers were carried out using 1 to 1,000 dilutions. The microbial source next to three sequential batch cultures (transfers 1, 3 and 10) were analyzed by bacterial 16S rRNA gene and fungal ITS1 pyrosequencing. Faith’s phylogenetic diversity values became progressively smaller from the inoculum to the sequential batch cultures. Moreover, increases in the relative abundances of Enterobacteriales*,* Pseudomonadales*,* Flavobacteriales and Sphingobacteriales were noted along the enrichment process. Operational taxonomic units affiliated with *Acinetobacter johnsonii*, *Pseudomonas putida* and *Sphingobacterium faecium* were abundant and the underlying strains were successfully isolated. Interestingly, *Klebsiella variicola* (OTU1062) was found to dominate in both consortia, whereas *K. variicola*-affiliated strains retrieved from untreated wheat straw consortia showed endoglucanase/xylanase activities. Among the fungal players with high biotechnological relevance, we recovered members of the genera *Penicillium*, *Acremonium*, *Coniochaeta* and *Trichosporon*. Remarkably, the presence of peroxidases, alpha-L-fucosidases, beta-xylosidases, beta-mannases and beta-glucosidases, involved in lignocellulose degradation, was indicated by predictive bacterial metagenome reconstruction. Reassuringly, tests for specific (hemi)cellulolytic enzymatic activities, performed on the consortial secretomes, confirmed the presence of such gene functions.

**Conclusion:**

In an in-depth characterization of two wheat straw degrading microbial consortia, we revealed the enrichment and selection of specific bacterial and fungal taxa that were presumably involved in (hemi) cellulose degradation. Interestingly, the microbial community composition was strongly influenced by the wheat straw pretreatment. Finally, the functional bacterial-metagenome prediction and the evaluation of enzymatic activities (at the consortial secretomes) revealed the presence and enrichment of proteins involved in the deconstruction of plant biomass.

## Background

Efficient bioconversion of lignocellulosic substrates depends critically on the functioning of multispecies microbial consortia rather than single strains [1]. In such consortia, secretion of the enzymes involved in biodegradation, as affected by the interactions between the microbial players (bacteria-fungi), is of crucial importance [2,3]. Wheat straw, as the source of lignocellulose, can potentially serve to provide building blocks for production of plastics or energy in biofuels [4]. The conversion of lignocellulosic polymers into monomers that can be further processed involves the synergistic action of a range of secreted enzymes, that is, peroxidases, xylanases and endo/exoglucanases [5,6]. In spite of the fact that intricate knowledge on the decomposition process is lacking, many bacteria are known to be capable of producing such enzymes. In particular, members of the Gammaproteobacteria, Firmicutes and Bacteroidetes have been implicated in lignocellulose biodegradation [7,8]. Moreover, fungi like *Trichosporon* and *Coniochaeta* are considered as potential sources of hydrolytic enzymes, in particular those involved in the bioconversion of (toxic) furanic compounds and in the production of unique secondary metabolites [9,10]. In addition, recent evidence suggests that, from the biotechnological perspective, *Penicillium*, *Acremonium* and *Trichoderma* species represent fungi that are applicable in the production of commercial lignocellulases [11].

The current literature indicates several strategies by which effective microbial consortia can be obtained [12]. In addition, the construction of target microbial communities can be aided using stable isotope probing (SIP) [13]. However, SIP suffers from drawbacks related to cross-feeding phenomena and/or the possible detection of bacterial or fungal predators of labeled cells, that is, those representing “microbial cheaters” [14]. Thus, a valid strategy to obtain efficient microbial consortia that degrade lignocellulosic matter is *ex situ* dilution to stimulation, using (partially unlocked) plant material as the unique energy and carbon source [15,16]. Due to selective processes, this last approach results in a stimulus of (biodegradation) function within the emerging consortia during succession [12]. The enrichment cultures produced can then provide a robust platform for biotechnological applications [17-19].

Unfortunately, cultivation-based analyses of complex microbial consortia are restrictive, as key organisms may be omitted. Thus, DNA-based high-throughput sequencing techniques have been recently applied to lignocellulolytic consortia [20,21]. The studies performed so far have, however, only addressed the role of bacteria, to the exclusion of fungal players. Fungi, either in the mycelial or yeast form, can have dominant roles in lignocellulose decomposition in plant litter and soil [22,23]. In lignocellulosic enrichment cultures, the bacterial and fungal diversities may be driven by the microbial source, available substrates, pH, redox potential, temperature and possible toxic compounds [24-26]. Thus, such consortia need to be assessed over time in relation to conditions and metabolic fluxes among key members, which is important for further “consortium engineering” [2].

The classical bacterial 16S rRNA gene and fungal ITS1 based markers are useful to describe community composition but do not provide information on the genes that are involved in lignocellulose deconstruction. Recently, Langille *et al*. (2013) [27] suggested a way to overcome such a limitation. They developed the software PICRUSt (Phylogenetic Investigation of Communities by Reconstruction of Unobserved States) to predict the occurrence of functions in microbial communities solely on the basis of bacterial 16S rRNA gene sequences. Although such an approach is theoretically fraught with uncertainties, realistic predictions of function in low-complexity environments were given. Thus, PICRUSt has been used to analyze the human intestinal mucosal surface microbiome, and the results correlated fairly well with the extant metabolome, suggesting a relationship between inferred function and metabolites found [28]. However, the method needs extreme caution in the interpretation of its outcomes, given the known impact of horizontal gene transfer (HGT) across the genomes of the members of most microbial communities. In addition, the quality of these functional predictions is largely dependent on the availability of annotated reference genomes.

In a previous study [29], we reported the construction of two novel bacterial-fungal consortia involved in the bioconversion of lignocellulose next to furanic compounds. We described their characteristics based on bacterial cell counts, quantitative PCR (qPCR), denaturing gradient gel electrophoresis (DGGE) analyses and isolation of some key consortium members. In addition, we designed a novel iodide oxidation method to detect 5-hydroxymethylfurfural oxidoreductase activity. In the current study, we expanded our previous work by focusing on the metataxonomic evaluation (based on bacterial 16S rRNA gene and fungal ITS1 pyrosequencing data) of two lignocellulolytic microbial consortia enriched on untreated versus pretreated wheat straw. We here analyze the successional microbial diversity and community composition of the two consortia and apply PICRUSt, thus predicting genes for functions involved in lignocellulose metabolism. Moreover, we evaluated the joint expression of some of these genes in the secretome, by quantification of specific (hemi)cellulolytic enzymatic activities. The two consortia constitute starting points for biotechnological applications in the light of their possible capacities in the conversion of lignin, (hemi)cellulose, furanic compounds and cello-oligosaccharides.

## Results

### Analysis of the community structures and diversities of two microbial consortia

**Figure 1:**
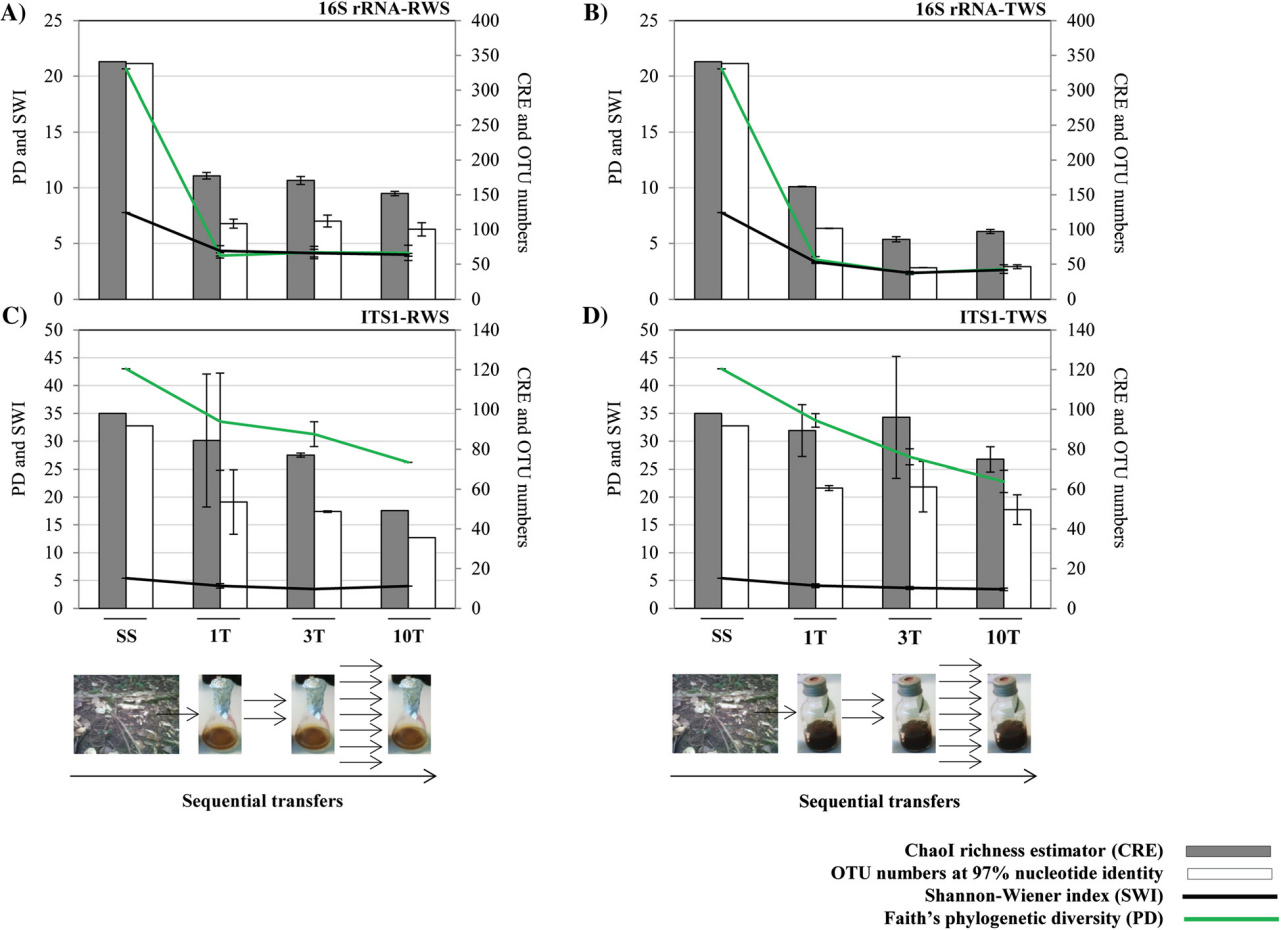
**Diversity indices in the soil inoculum (SS) and in enriched cultures (RWS and TWS) along the sequential batches.** Diversity indices and richness estimator measured using **(A, B)** rarefied bacterial 16S rRNA and **(C, D)** ITS1 region sequences. Bars refer to standard errors (n = 2). For SS and 10-RWS (ITS1) only one sample was analyzed. The arrows represent the number of parallel sequential transfers between 1T (transfer 1), 3T (transfer 3) and 10T (transfer 10) (for more detail see Methods).

Overall, 18,200 trimmed-rarefied sequences of bacterial 16S rRNA from the forest soil inoculum (SS) as well as the RWS (untreated wheat straw) and TWS (heat-treated wheat straw) consortia (n = 13) were analyzed. The rarefied sequencing data (1,400 sequences per sample) were binned into 338, 109 (±6.5) and 102 (±0.3) abundant operational taxonomic units (OTUs), for SS, RWS and TWS (both at transfer 1 - T1), respectively (Figure 1A and B; Additional file 1). At T10 (transfer 10, approximately 70 days after setting up the first microcosm), we observed the presence of 100 (±9.5) and 47 (±2.7) OTUs for RWS and TWS, respectively.Based on the bacterial 16S rRNA gene sequences, the Faith’s phylogenetic diversity (PD) and Chao richness estimator (CRE) values in SS were 20.66 and 340.90, respectively. In contrast, these values (at T10) in RWS were 4.15 (±0.68) and 151.87 (±3.09). For RWS, in particular the CRE values for 16S rRNA gene decreased slightly from T1 to T10. However, the PD and Shannon-Wiener index (SWI) values did not show large changes, for example with PD values of 3.91 (±0.23) and 4.15 (±0.68) for T1 to T10, respectively (Figure 1A). For TWS, the bacterial consortia also showed progressively decreasing CRE and PD values, with the higher ones in the SS and T1 (161,67 ± 0.49 and 3.54 ± 0.26 for CRE and PD, respectively) (Figure 1B).For fungal communities across all samples, 6,600 trimmed-rarefied sequences (n = 12) were analyzed. One sample (T10 in RWS) was omitted due to low-quality reads. At the sequencing depth of 550 sequences per sample, 91, 54 (±16) and 61 (±1.2) different OTUs were identified in SS, T1/RWS and T1/TWS, respectively (Figure 1C and D). At T10, 36 and 50 (±7.4) OTUs were identified for RWS and TWS, respectively. In SS, the fungal consortia showed values of 43.02, 97.95 and 5.41 for PD, CRE and SWI, respectively. For RWS, the PD values were 33.52 (±8.71) and 26.20 (at T1 and T10, respectively) (Figure 1C). As expected, we observed a decrease of the PD values from SS (43.02) to T10 (22.81 ± 2.00). The CRE values also showed a decrease, from 97.95 in SS to 89.33 (±13.02) at T1 and 74.93 (±6.28) at T10 (Figure 1D).

### Relative abundance of bacterial and fungal types in the microbial consortia

**Figure 2:**
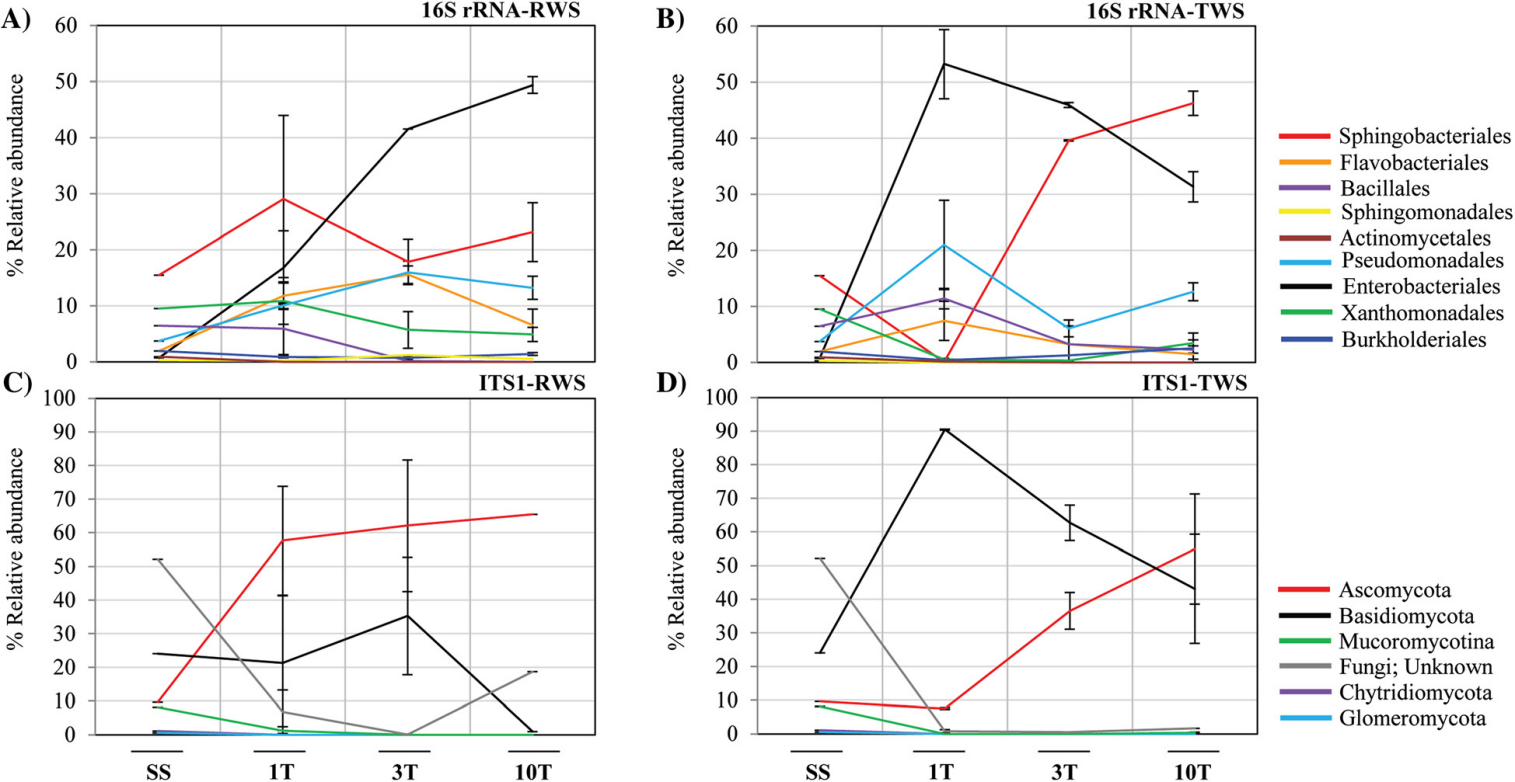
**Relative abundance (bacteria and fungi) in the soil inoculum (SS) and in enriched cultures (RWS and TWS) along the sequential batches.** Relative abundance (%) of the most abundant **(A, B)** bacterial orders and **(C, D)** fungal phylum members based on 1,400 (16S rRNA) and 550 (ITS1) sequences.

The source (SS) bacterial community was predominantly composed of Acidobacteria (19.28%), Gammaproteobacteria *(*18.07%), Bacteroidetes (17.5%) and Betaproteobacteria (10.21%) (Additional file 2A). For RWS at T10, increases of the relative abundances (RA) of Gammaproteobacteria and Bacteroidetes (67.64% ± 2.28 and 29.78% ± 2.28, respectively) were noted. For TWS, the RA of Gammaproteobacteria was high at T1 (80.5% ± 2.14), declining at T10 to 47.42% (±2.5) (Additional file 2A). Successive subcultivation led to the enrichment of OTUs mainly affiliated with nine bacterial orders (Figure 2 and Additional file 2B). For RWS, we observed an increase of the RA of Enterobacteriales from 0.71% in SS to 49.39% (±1.46) in T10. Pseudomonadales and Flavobacteriales showed a similar behaviour, with an increase of the RA from SS (3.71% and 1.92%, respectively) to T3 (15.96% ± 2.03 and 15.57% ± 1.57, respectively), with a small subsequent reduction in T10 (Figure 2A). For TWS, the RA of Enterobacteriales increased to 53.21% (±6.14) in T1, decreasing along the transfers to 31.32% (±2.67) in T10 (Figure 2B). In contrast, the Sphingobacteriales RA showed an opposite pattern, starting at 15.5% in SS and decreasing to 0.03% (±0.03%) at T1, with an increase to 46.25% (±2.17) at T10. Members of Bacillales and Flavobacteriales showed a similar tendency in TWS, with higher values at T1 and lower ones at T10. Pseudomonadales was the third most abundant order, with values of 12.60% (±1.60) and 20.96% (±7.96) at T1 and T3, respectively (Figure 2B).

The most abundant fungi in SS belonged to unclassified types (possibly uncultured Ascomycota) (52.18%), followed by Basidiomycota (24%), Ascomycota (9.63%), Mucoromycotina (8.18%) and Chytridiomycota (1.09%). Totals of 24, 172 and 97 sequences in SS, RWS and TWS, respectively, showed no affiliation against the UNITE and/or GenBank databases, and were removed from the analyses (Additional file 3A). For RWS, the RA of Ascomycota and Basidiomycota increased compared to that in SS. However, the replicate patterns were internally not very consistent (Figure 2C). On the other hand, TWS showed high consistency between replicates in each transfer (Additional file 3A). The RA of Basidiomycota was 90.45% (±0.09) in T1, decreasing along the transfers to 43.09% (±16.18) in T10. In contrast, the RA of Ascomycota increased from 7.54% (±0.27) in T1 to 54.09% (±16.18) in T10 (Figure 2D).

### Relative abundance at genus level and structure of the microbial consortia

**Figure 3:**
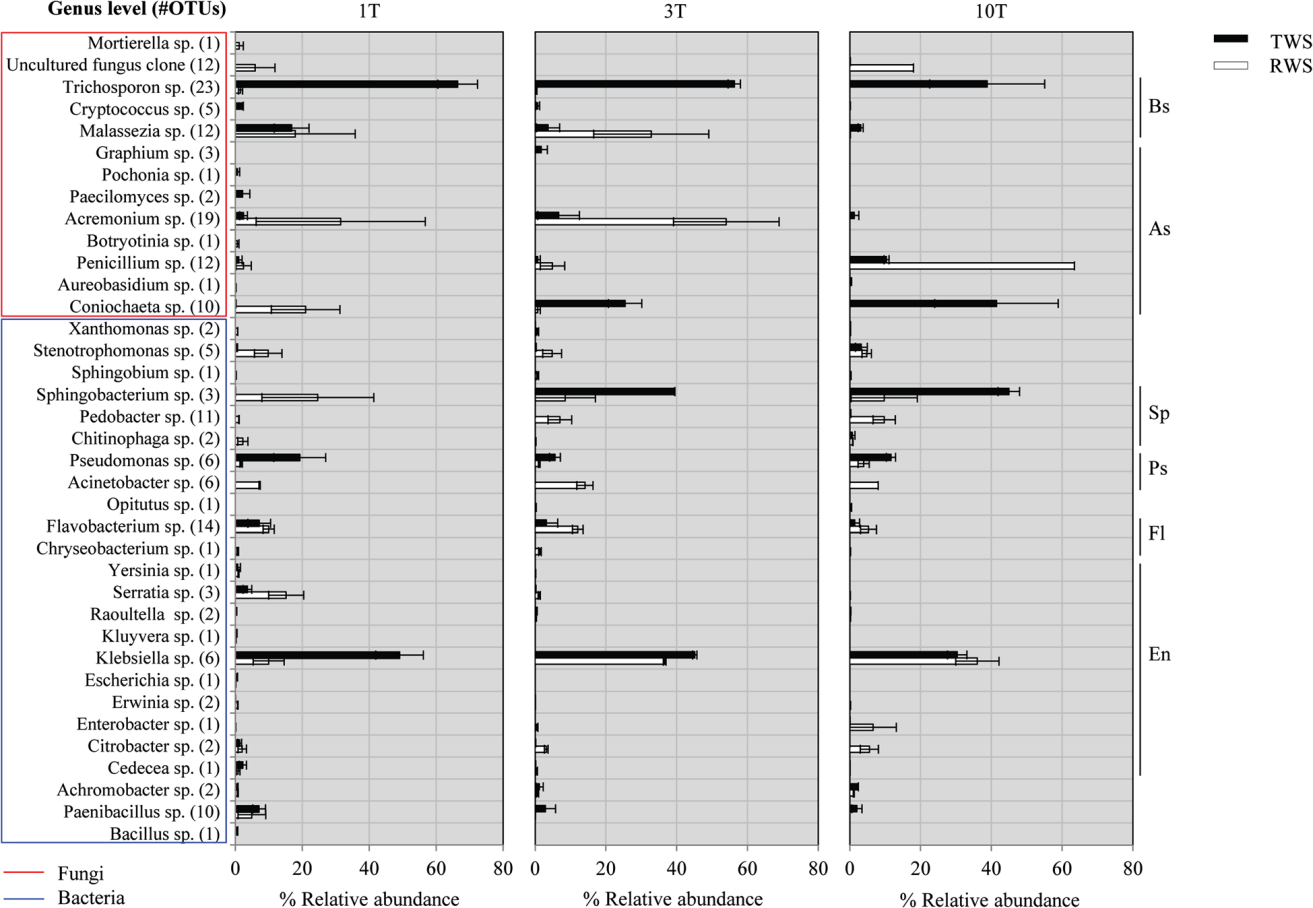
**Relative abundances of the most abundant genera in the sequential batches enriched cultures (RWS and TWS).** Abbreviations: Basidiomycota (Bs), Ascomycota (As), Sphingobacteriales (Sp), Pseudomonadales (Ps), Flavobacteriales (Fl), Enterobacteriales (En).

To assess the RA of each genus in the RWS and TWS microbial consortia, we removed the least abundant OTUs (those containing less than 10 sequences in total). We thus used 92.64% (±1.50) to 98.50% (±0.57), and 81.63% (±0) to 95.65 (±1.09) of the 1,400 16S rRNA gene and 550 ITS1 sequences, respectively. On this basis, 24 bacterial and 13 fungal genera were detected across all samples in both enrichment strategies (omitting the source SS) (Figure 3). The bacterial communities at T1 for RWS and TWS showed eight abundant genera, defined as having an RA > 5%; these were *Stenotrophomonas*, *Sphingobacterium*, *Acinetobacter*, *Flavobacterium*, *Pseudomonas*, *Serratia*, *Klebsiella* and *Paenibacillus*.

After the third transfer (T3), when communities had stabilized, the bacterial genera in RWS with the highest RA were *Klebsiella* (about 35%), *Acinetobacter* (about 12%), *Flavobacterium* (about 9%), *Sphingobacterium* (about 9%), *Pedobacter* (about 8%) and *Enterobacter* (about 3%). This was followed by *Stenotrophomonas*, *Citrobacter*, *Sphingobium* and *Chitinophaga* (1 to 2%). For TWS, the most abundant bacterial genera at T3 and T10 were *Sphingobacterium* (about 42%), *Klebsiella* (about 38%) and *Pseudomonas* (about 8%), whereas the least abundant OTUs (1 to 2%) were affiliated with S*tenotrophomonas*, *Flavobacterium*, *Achromobacter* and *Paenibacillus* species*.* Concerning the fungi, for RWS the genera with highest RA at T1 and T3 were *Acremonium* (about 42%), *Malassezia* (about 25%) and *Coniochaeta* (~10%), whereas at T10 we observed highest RA for *Penicillium* (about 63%). For TWS, *Trichosporon* (66.45% ± 5.90) and *Malassezia* (16.81% ± 5.18) were most abundant at T1. After this stage, we observed an increase of the RA of *Coniochaeta* (about 33%), *Penicillium* (about 5%) and *Acremonium* (about 4%). In addition, the RA of *Trichosporon* (about 39%) was also high at T10 (Figure 3).

**Figure 4:**
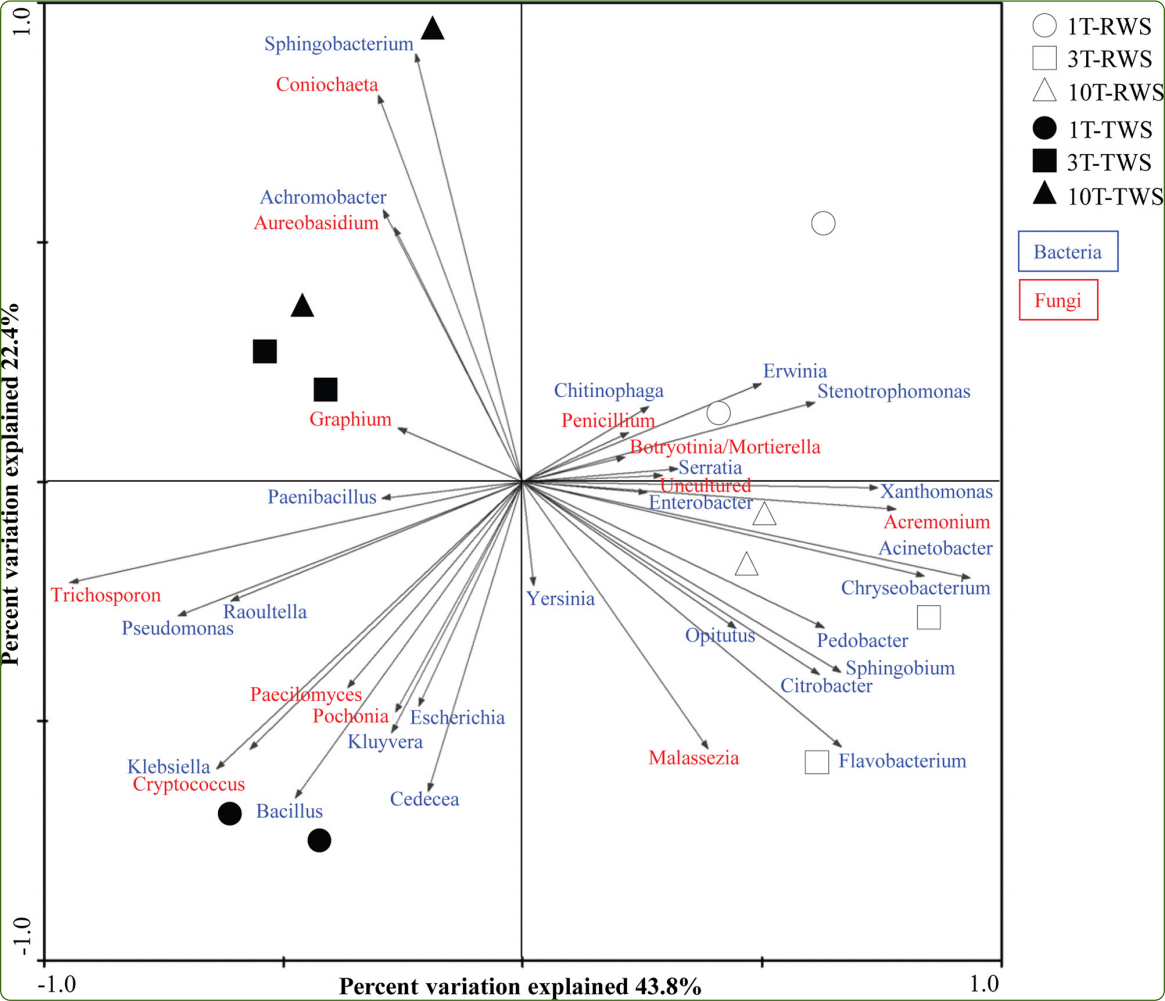
Principal components analysis (PCA) of the most abundant genera in the enriched cultures (RWS and TWS).

A principal components analysis (PCA) of the data showed that *Flavobacterium*, *Acinetobacter*, *Pedobacter*, *Citrobacter*, *Chryseobacterium*, *Opitutus*, *Sphingomonas*, *Acremonium* and *Malassezia* were preferentially selected at T10 for RWS, while *Sphingobacterium*, *Achromobacter*, *Coniochaeta* and *Aureobasidium* were increased at T10 for TWS (Figure 4).

### Bacterial OTUs related to (hemi)cellulolytic strains

**Figure 5:**
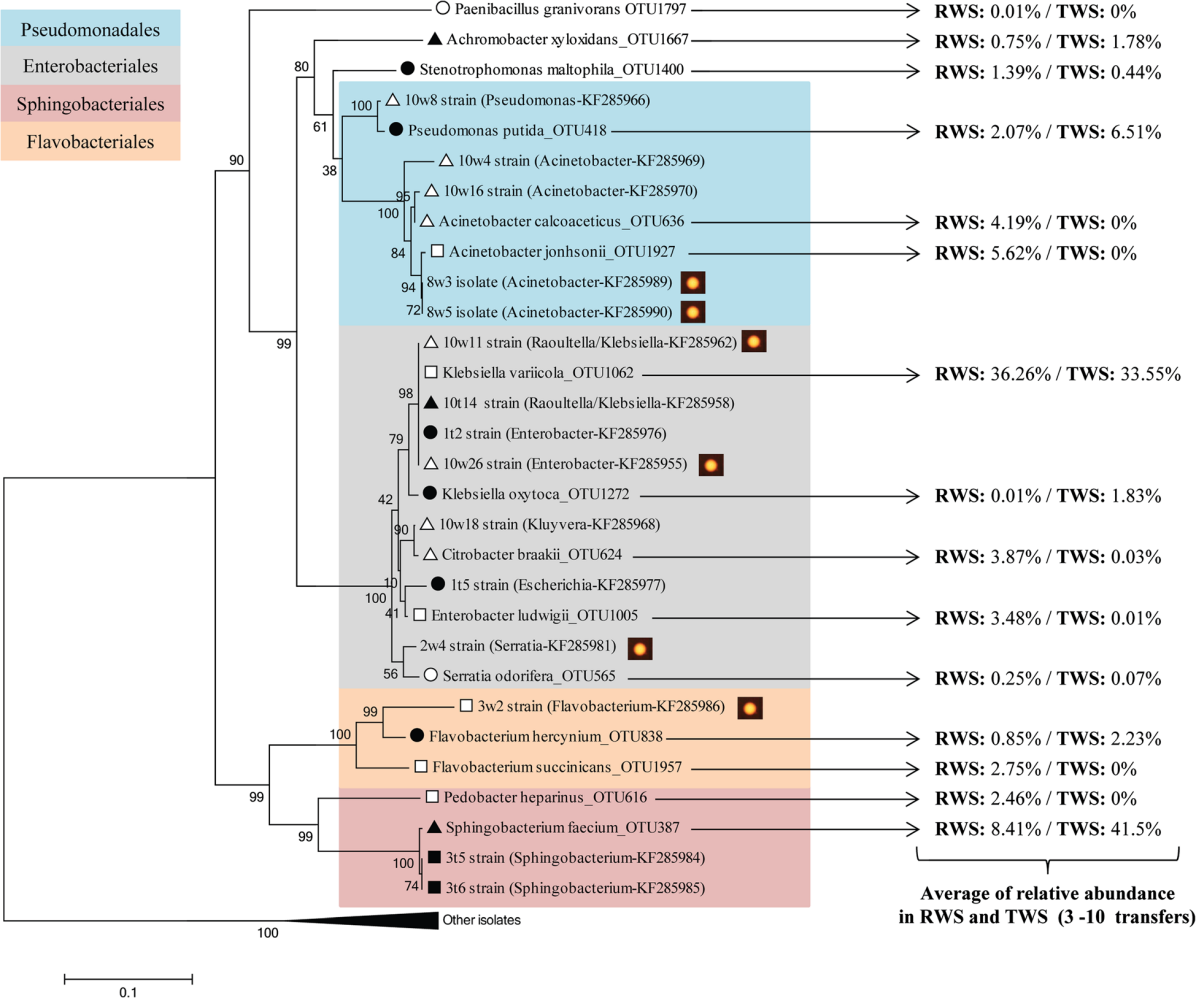
**Neighbour-joining phylogenetic tree of partial bacterial 16S rRNA gene sequences (274 nucleotides) from bacterial strains and most abundant OTUs in the enriched cultures (RWS and TWS).** Yellow dots represent (hemi)cellulolytic activity in agar plates (CMC-ase and xylanase). Taxonomic affiliation and accession numbers of isolates from the GenBank are shown in parentheses. Right side shows average relative abundance of each OTU in the 3 to 10 transfers. Circles, squares and triangles represent sequences retrieved in T1, T3 and T10, respectively in RWS (white) and TWS (black).

A total of 10 out of the 15 abundant bacterial OTUs detected by direct molecular assessment was recovered as isolates (Figure 5). Bacterial isolates were recovered by dilution plating on R2A agar and presumptively identified using 16S rRNA gene sequencing [29]. Among these, non-(hemi)cellulolytic strain 10w8 isolated from TWS matched *Pseudomonas putida*_OTU418 (99% identity). In RWS, two OTUs representing the genus *Acinetobacter* were found to be abundant. Sequence-wise, the isolated strains 8w3 and 8w5, which were found to have endoglucanase and xylanase activities, matched one OTU, affiliated with *Acinetobacter johnsonii* (OTU1927), whereas strain 10w16, which was devoid of any (hemi)cellulolytic activity (based on its activity on carboxymethylcellulose (CMC) and xylan from birchwood), matched *Acinetobacter calcoaceticus*_OTU636. The sequence of OTU1062 (*Klebsiella variicol*a) represented the most abundant OTU in RWS (approximately 37%), and the RWS- and TWS-derived strains 10w11, 10w26, 10 t14 and 1 t2 matched this sequence type. Interestingly, the strains retrieved from RWS (10w11 and 10w26) showed (hemi)cellulolytic activity, whereas those from TWS (10 t14 and 1 t2) did not (Figure 5). In accordance with the phylogenetic tree and low identity (90%), we not consider that *Flavobacterium hercynium*_OTU838 represents the (hemi)cellulolytic bacterial strain 3w2. Moreover, strains 3 t5 and 3 t6, which likely represented the highly abundant *Sphingobacterium faecium*_OTU387, did not show CMC-ase and xylanase activities on agar plates.

### Predicting functions involved in lignocellulose deconstruction

Totals of 25 and 17 genes related with plant biomass deconstruction were predicted to be consistently enriched from the SS to the RWS and TWS consortia at T10, respectively. Conversely, 34 and 18 predicted genes were enriched from the sequential batches (T1 to T10) in RWS and TWS, respectively (Table 1). Interestingly, predicted genes that codify for glycolate oxidase (EC:1.1.3.15), alpha-L-fucosidase (EC:3.2.1.51), alpha-N-arabinofuranosidase (EC:3.2.1.55), endo-1.4-beta-xylanase (EC:3.2.1.8), alpha-L-rhamnosidase (EC:3.2.1.40) and maltooligosyltrehalose trehalohydrolase (EC:3.2.1.93) decreased in number along the sequential transfers in RWS; however, they increased in TWS (Table 1).

**Table 1:** Selection of 40 genes involved in lignocellulose degradation and average number of predicted genes by PICRUSt

				**Transfer in RWS**			**Transfers in TWS**		
	**KEGG gene description [EC number]**	**CAZy family, GH or AA**	**SS**	**1 T**	**3 T**	**10 T**	**1 T**	**3 T**	**10 T**
**Lignin**	Glycolate oxidase [EC:1.1.3.15]	AA7	374	127	63	62	119	192	231^d^
	Catalase [EC:1.11.1.6]	AA2	253	417^b^	339^b^	578^bc^	384^b^	486^b^	521^bd^
	Vanillate monooxygenase [EC:1.14.13.82]	NC	102	94	92	230^bc^	70	41	55
	Catalase/peroxidase [EC:1.11.1.6 1.11.1.7]	AA2	656	349	351	670^bc^	343	310	307^d^
	Glutathione peroxidase [EC:1.11.1.9]	AA2	536	477	400	651^bc^	371	468	513^d^
	Cytochrome c peroxidase [EC:1.11.1.5]	AA2	468	287	210	301^c^	69	310	326^d^
	Chloride peroxidase [EC:1.11.1.10]	AA2	410	236	200	410^c^	212	258	250^d^
	Thiol peroxidase. atypical 2-Cys peroxiredoxin [EC:1.11.1.15]	AA2	93	212^b^	174^b^	351^bc^	216^b^	252^b^	237^bd^
	Peroxiredoxin (alkyl hydroperoxide reductase subunit C) [EC:1,11,1,15]	AA2	427	349	343	605^bc^	377	351	333
**(Hemi)cellulose**	Alpha-amylase [EC:3.2.1.1]	GH (13, 14, 57, 119)	55	133^b^	190^b^	546^bc^	234^b^	145^b^	95^b^
	Alpha-galactosidase [EC:3.2.1.22]	GH (4, 27, 32, 36, 57, 97, 110)	605	198	159	404^c^	86	183	198^d^
	Alpha-L-fucosidase [EC:3.2.1.51]	GH (29, 95)	927	1150^b^	456	409	30	1489^b^	1604^bd^
	Alpha-mannosidase [EC:3.2.1.24]	GH (31, 38, 92)	181	171	140	272^bc^	190^b^	265^b^	227^bd^
	Alpha-N-arabinofuranosidase [EC:3.2.1.55]	GH (3, 10, 43, 51, 54, 62)	667	320	207	267	128	339	361^d^
	Arabinogalactan endo-1.4-beta-galactosidase [EC:3.2.1.89]	GH (53)	24	32^b^	37^b^	190^bc^	39^b^	27^b^	24
	Beta-galactosidase [EC:3.2.1.23]^a^	GH (1, 2, 3, 35, 42, 50)	940	554	403	842^c^	243	674	688^d^
	Beta-glucuronidase [EC:3.2.1.31]	GH (1, 2, 79)	192	12	26	35^c^	13	4	4
	Beta-mannosidase [EC:3.2.1.25]^a^	GH (1, 2, 5)	162	16	32	34^c^	23	13	16
	Carboxylesterase [EC:3.1.1.1]	GH (5)	26	6	3	10^c^	55^b^	15	29^b^
	Endo-1.4-beta-xylanase [EC:3.2.1.8]^a^	GH (5, 8, 9, 10, 11, 12, 16, 30, 43, 44)	362	161	92	74	41	153	176^d^
	Evolved beta-galactosidase subunit alpha [EC:3.2.1.23]^a^	GH (1, 2, 3, 35, 42, 50)	1	14^b^	17^b^	125^bc^	13^b^	3 ^b^	4 ^b^
	Levanase [EC:3.2.1.65]	GH (32)	75	119^b^	69	181^bc^	27	139^b^	149^bd^
	Lysophospholipase [EC:3.1.1.5]	GH (5)	59	91^b^	121^b^	283^bc^	151^b^	98^b^	61^b^
	Mannan endo-1.4-beta-mannosidase [EC:3.2.1.78]^a^	AA10- GH (5, 9, 26, 44, 113)	8	8	21^b^	29^bc^	5	2	1
	Xylan 1,4-beta-xylosidase [EC:3.2.1.37]^a^	GH (1, 3, 30, 39, 43, 52, 54, 116, 120)	281	122	132	412^bc^	120	63	57
	Endoglucanase [EC:3.2.1.4]^a^	GH (5–9, 12, 16, 44, 45, 48, 74, 124)	472	192	193	474^bc^	203	130	117
**Cellobiose**	6-phospho-beta-glucosidase [EC:3.2.1.86]^a^	GH (1, 4)	54	414^b^	607^b^	1842^bc^	783^b^	514^b^	333^b^
	Alpha-glucosidase [EC:3.2.1.20]	GH (4, 13, 31, 63, 97, 122)	774	415	359	702^c^	258	418^c^	411^d^
	Beta-glucosidase [EC:3.2.1.21]^a^	GH (1, 3, 5, 9, 30, 116)	1468	771	752	1786^bc^	671	783	754^d^
	Glucan endo-1.3-beta-D-glucosidase [EC:3.2.1.39]^a^	GH (16, 17, 55, 64, 81, 128)	4	9^b^	24^b^	29^bc^	5^b^	2	1
	Oligo-1.6-glucosidase [EC:3.2.1.10]^a^	GH (13, 31)	106	13	21	28^c^	11	4	6
**Cello-oligosaccharides**	Beta-fructofuranosidase [EC:3.2.1.26]	GH (32, 68, 100)	146	108	166^b^	642^bc^	217^b^	146	103
	Glyceraldehyde 3-phosphate dehydrogenase [EC:1.2.1.12]	NC	542	714^b^	583^b^	1033^bc^	490	840^b^	833^bd^
	Alpha.alpha-trehalase [EC:3.2.1.28]	GH (13, 15, 37, 65)	210	277^b^	282^b^	596^bc^	278^b^	314^b^	265^b^
	Trehalose-6-phosphate hydrolase [EC:3.2.1.93]	GH (13)	18	82^b^	112^b^	306^bc^	161^b^	106^b^	60^b^
	Maltose-6′-phosphate glucosidase [EC:3.2.1.122]	GH (4)	16	114^b^	158^b^	504^bc^	220^b^	140^b^	87^b^
	Alpha-L-rhamnosidase [EC:3.2.1.40]	GH (13, 78, 106)	140	303^b^	117	105	10	405^b^	435^bd^
	Maltooligosyltrehalose trehalohydrolase [EC:3.2.1.141]	GH (13)	225	110	45	50	55	154	186^d^
	PTS system. cellobiose-specific IIB component [EC:2.7.1.69]	NC	36	300^b^	407^b^	1527^bc^	500^b^	307^b^	196^b^
	PTS system. cellobiose-specific IIC component	NC	59	447^b^	587^b^	1963^bc^	819^b^	522^b^	335^b^

The absolute values show the number of predicted genes per each 1,000 rarefied 16S rRNA sequences analyzed (normalized data).

^**a**^Enzymatic activities detected in the secretome by MUF-substrate quantification.

^**b**^Enriched functions in RWS and TWS (compared with the soil inoculum SS).

^**c**^Enriched functions in 10 T-RWS (compared with 1 T-RWS).

^**d**^Enriched functions in 10 T-TWS (compared with 1 T-TWS).

NC) Not classified.

GH) Glycosyl hydrolase.

AA) Auxiliary activities.

**Figure 6:**
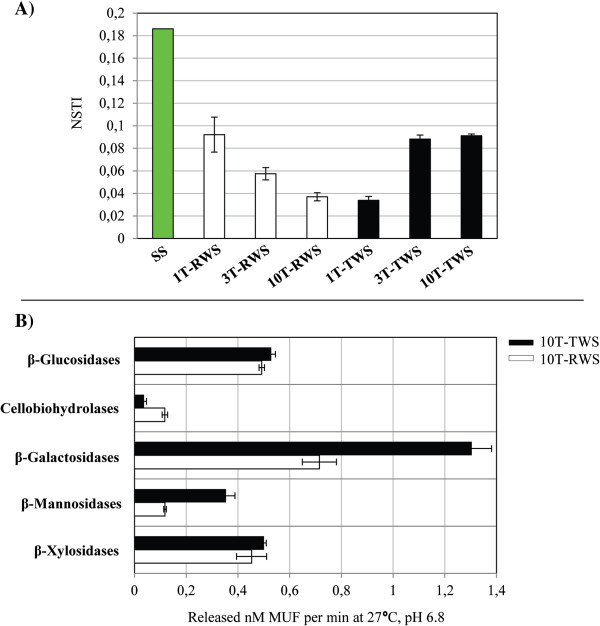
**NSTI values and quantification of enzymatic activities by methylumbelliferyl (MUF)-substrates. (A)** NSTI values in the soil inoculum (SS) and in enriched cultures (RWS and TWS) along the sequential batches. **(B)** Quantification of specific enzymatic activities by MUF-substrates in the consortial secretome of the last transfer in both enriched cultures.

Six predicted genes potentially related to lignin bioconversion were enriched in RWS at T10, while one (catalase - EC:1.11.1.6) was enriched in TWS at T10, compared with the SS source (Table 1). With respect to (hemi)cellulose bioconversion, the number of predicted genes that encode alpha-mannosidase (EC:3.2.1.24) and levanase (EC:3.2.1.65) increased along the sequential transfers (T1 to T10) in both cultures and also were higher than those in the SS source. The predicted genes that encode glucosidase (alpha and beta) enzymes also increased along the sequential transfer in both strategies. Moreover, many predicted genes that were already abundant in SS, such as those that encode beta-galactosidases (EC:3.2.1.23) and beta-glucosidases (EC:3.2.1.21), showed an extra increase by the sequential transfer in both cultures. Also, xylan 1.4-beta-xylosidase (EC:3.2.1.37) and endoglucanase (EC:3.2.1.4) related predicted genes were either enriched or depleted along the sequential transfers in RWS and TWS, respectively (Table 1). In addition, a beta-mannosidase (EC:3.2.1.25) related gene was found in both enrichment cultures. Regarding the accuracy of metagenome predictions, the Nearest Sequenced Taxon Index (NSTI), which quantifies the uncertainty of the prediction (lower values mean a better prediction), decreased from 0.18 in SS to 0.03 (RWS) and 0.09 (TWS) at T10 (Figure 6A). The NSTI metric represents the sum of phylogenetic distances for each organism in the OTU table to its nearest relative with a sequenced reference genome, measured in terms of substitutions per site in the 16S rRNA gene and weighted by the frequency of that organism in the OTU table [27].

### Quantification of specific enzymatic activities related to (hemi)cellulose bioconversion

As shown by direct enzymatic assays, beta-xylosidases, beta-galactosidases, beta-mannosidases, cellobiohydrolases and beta-glucosidases were active in the secretome of both consortia. Interestingly, we observed high beta-mannosidase (0.35 nM 4-methylumbelliferyl (MUF)/min) and beta-galactosidase (1.3 nM MUF/min) activities in TWS but low ones in RWS. In addition, cellobiohydrolases showed higher activities in RWS (0.11 nM MUF/min) compared to TWS (0.03 nM MUF/min). Beta-galactosidases, beta-glucosidases and beta-xylosidases also showed higher activity values in both secretomes compared with the other two enzymes (Figure 6B). The bacterial abundance at T10 was evaluated by cell counts, and we observed increases from the inoculum level, around 5, to about 8.4 log bacterial cells/mL in both consortia (data not shown).

## Discussion

The use of microbial consortia in lignocellulose transformation is likely to reduce impairments in bioprocesses using lignocellulosic matter, such as incompletely synergistic enzymes, pH regulation, the presence of toxic compounds, end-product inhibition and tolerance to environmental fluctuations [2,18]. Previously, several different types of plant biomass, such as sugarcane bagasse, poplar wood chips and switchgrass [15,30,31], have been used in the selection of biodegradative microbial consortia. In our work, wheat straw, either torrefied (pretreated with heat at 240°C) or not, was used. However, torrefaction, which is proposed as a valuable step in waste plant biomass valorization [32], introduces furanic compounds and/or cello-oligosaccharides in the medium [24]. Other studies suggested that lignocellulose-degrading soil communities are best addressed using SIP analysis based on ^13^C-substrates [33], whereas lignin-degradative microbial communities can be enriched directly in soil [34].

Lignocellulolytic microbes are important members of forest soil communities and aid in the degradation of litter [35,36]. Our dilution-to-stimulation approach from a forest soil source community resulted in a stimulus of biodegradative function within the emerging bacterial-fungal consortia. Moreover, qPCR showed that the bacterial 16S rRNA gene copy numbers were higher than the fungal ITS1 copy numbers (about 2 log units) in both substrates [29]. Notably, the microbial diversity became markedly reduced in both consortia compared to the source SS (Figure 1), suggesting the selection of particular taxa (including lignocellulose and possibly cello-oligosaccharide eaters) at the detriment of others. On the other hand, the apparently low microbial richness in SS could be related to the deletion of all singletons in our bioinformatic analysis. The bacterial CRE values decreased along the sequential batches, indicating enrichment of OTUs that grew consistently well in the enrichment. Interestingly, the TWS consortia showed low bacterial diversities compared to the RWS ones, possible due to the presence of toxic compounds such as furanic aldehydes. With respect to fungal diversity and richness, our data showed values that were similar between RWS and TWS, indicating that substrate type did not strongly affect fungal diversity. However, Faith’s PD measure suggested the selection of particular lignocellulolytic fungi in both consortia. Moreover, both UniFrac unweighted distances and PCA (Figure 4; Additional file 4) showed that the consortia were highly influenced by substrate type (variation explained: 43.8%), which is similar to other reported data [21,37]. In addition, the structures of both consortia became less different after T3. In our previous analysis based on PCR-DGGE, stability of the dominant microorganisms after six transfers was reported [29].

The enrichment process increased the abundances of the bacterial orders Enterobacteriales, Pseudomonadales, Flavobacteriales and Sphingobacteriales (Figure 2A and B). In 2012 Eichorst and Kuske [38] performed SIP using ^13^C-maize cellulose to evaluate the biodegradative microbial communities in different soils. They identified Burkholderiales, Caulobacterales, Rhizobiales, Sphingobacteriales and Xanthomonadales as the active bacterial groups. Enterobacteriales and Pseudomonadales were also enriched in insect herbivore microbiomes and lignin-enriched cultures, respectively [39,40]. Interestingly, in our RWS consortia the abundance of Enterobacteriales increased after T1, but the number of OTUs decreased, suggesting the selection of specific strains, for example, *K. variicola*, within this group. A similar pattern was observed for Sphingobacteriales in TWS, although the number of OTUs did not change after T3 (Additional file 5).

In soils, Ascomycota may be highly abundant in early stages of litter decomposition, whereas particular basidiomycetous yeasts increase in the later stages, possibly due to their capacity to degrade more recalcitrant compounds [41]. Members of the orders Atheliales, Agaricales, Helotiales, Chaetothyriales and Russulales were found to be abundant in (coniferous) forest soils [35]. Here, we found a high abundance of Malasseziales and Hypocreales. Abundant fungal orders present in SS were even more enriched in RWS. In contrast, only low-abundance orders were enriched in TWS, such as Coniochaetales and Tremellales (Additional file 3B). Štursová *et al*. (2012) [22], using SIP analysis in soil and litter plant samples with ^13^C-cellulose, identified fungi affiliated with Dothideales, Leotiomycetes, Helotiales, Tremellales and Chaetothyriales. Thus, diverse fungi can be involved in lignocellulose bioconversion. We posit here that this diversity is dictated by the environmental source, substrate and methodology used for recovery and characterization.

Bacterial strains affiliated with an abundant *K. variicola*_OTU1062 showed (hemi)cellulolytic activity when isolated from RWS, but not from TWS, suggesting either HGT of mobile genes or differential gene expression between the isolates obtained from the two substrate types. Okeke and Lu (2011) [42] proposed that the capacity of *Klebsiella* types to degrade lignocellulose can be attributed to the acquisition of plasmids encoding (hemi)cellulolytic enzymes from the environment. However, Suen *et al*. (2010) [43] reported a chromosomal location in *K. variicola* At-22 of genes involved in plant biomass degradation, that is, beta-1,4-glucanase, alpha-xylosidases and alpha-mannosidases. Interestingly, the degradation of lignocellulose by an insect herbivore microbiome has been attributed to an association between *Leucoagaricus gongylophorus* (Basidiomycota) with *Klebsiella* species [39]. Another possible explanation for such findings arises from the regulatory mechanism of (hemi)cellulolytic genes, which is ultimately mediated by environmental conditions, in this case the torrefied substrate.

Members of *Citrobacter*, *Enterobacter*, *Acinetobacter*, *Pseudomonas*, *Flavobacterium* and *Stenotrophomonas* have the capacity to degrade plant lignin, (hemi)cellulose and/or CMC [44-48,40]. The presence of *Sphingobacterium* and *Pedobacter* in both microbial consortia may suggest the production of beta-glucosidases, indicating that these organisms are acting as “cheaters” that remove the cello-oligosaccharides produced by polymer degraders [49]. However, such organisms might also be involved in the production of aryl alcohol oxidases or endoxylanases [50,51]. We recently confirmed the production of beta-mannosidases, beta-galactosidases and beta-glucosidases by characterization of *S. faecium* (similar to OTU387, strain 3T5, data not shown). The presence of *Chryseobacterium*, *Opitutus*, *Chitinophaga* and *Xanthomonas* in RWS might relate to secondary functions, the nature of which is unclear. In TWS, members of *Stenotrophomonas*, *Pseudomonas* and *Flavobacterium* can be involved in the degradation of furanic compounds [52,29]. However, in both our consortia, *P. putida*_OTU418 might also act as a sugar cheater. Interestingly, Ronan *et al*. (2013) [53] reported an aerotolerant bacterial consortium composed of *Clostridium* and *Flavobacterium* that had the ability to produce ethanol. Moreover, the production of hydrogen by a consortium composed of *Clostridium*, *Klebsiella*, *Acinetobacter*, *Bacillus*, *Pseudomonas*, *Ruminococcus* and *Bacteroides* retrieved from sludge anaerobic digester has been evaluated [19]. These studies highlight the importance of aerobic bacterial members to deconstruct lignocellulose, such as those belonging to *Flavobacterium*, *Klebsiella* and *Acinetobacter*.

Concerning fungi, *Acremonium* is considered to be a very important organism for the production of (hemi)cellulases, as compared to *Trichoderma reesii*[54,55]. Moreover, *Penicillium* species have an elaborate enzymatic machinery to deconstruct lignocellulose, such as vanillyl-alcohol oxidases, copper-dependent polysaccharide monooxygenases [56], galactosidases, mannosidases and fucosidases [57]. In our consortia, the *Malassezia* species may have acted as sugar monomer cheaters, and their high abundances in RWS might be related with their high abundance in the SS. *Trichosporon* species are anamorphic basidiomycetous yeasts that are widespread in nature [58,59]. The presence of *Trichosporon* in the gut of xylophagous insects is probably facilitated by their ability to assimilate and transform lignin and various phenolic compounds [60]. Recent results from our group confirm the ability of our *Trichosporon* isolates to produce cellobiohydrolases and β-xylosidases (data not shown). *Trichosporon*, an oil-rich yeast, has high biotechnological potential and has been shown to be tolerant to furanic compounds [61,10]. It has been reported that the use of single fungal strains can be highly efficient to deconstruct specific compounds, such as lignin [62]. However, the breakdown of lignocellulose, for example, for biofuel production, often encounters great recalcitrance which will likely require the synergistic action of multispecies consortia (with higher gene diversity) to overcome it [2]. Some enzymatic transformations might be slow in such communities as a consequence of interspecific competition or even antagonism. To resolve these issues, enzyme cocktails that come from multispecies consortia may be retrieved and applied directly to the plant waste materials.

On theoretical grounds, one could bring up compelling evidence pointing to the scientific danger of attempting to link phylogeny with function by using PICRUSt, and the arguments extend to the limitation of current databases used in the software. However, the linkage might be regarded in a loose manner, including genes/functions that might be actually “floating” in the horizontal gene pool of the community. Thus, such functions are thought of as being not tightly linked to a phylogenetically determined species. In both microbial consortia, the uncertainty of the prediction as revealed by the NSTI was very reduced compared with that in the SS, thus indicating fair reliability and accuracy in the metagenome reconstruction (Figure 6A). The analysis predicted the enrichment of several genes in our consortia that were potentially involved in lignocellulose degradation, and also showed that TWS was possibly a poorer selector of such genes than RWS (Table 1). It was predicted that some peroxidases (EC:1.11.1-), classified as an “auxiliary activities” (AA2 family) in the CAZy (Carbohydrate-Active EnZymes database) [63], were enriched in both consortia by the sequential transfers. Such enzymes oxidize phenolic and non-phenolic aromatic compounds and can modify lignin polymers [56]. These enzymes were more evident in the RWS consortium, supporting its potential to act on lignin. Furthermore, glycolate oxidase (EC:1.1.3.15; an oxidoreductase capable of oxidizing glycolate to glyoxylate, producing reactive oxygen species) was progressively enriched in the TWS consortium, suggesting a correlation with the metabolism of furanic compounds. Glycolate oxidases are classified in CAZy as family AA7. In this family, we found gluco-oligosaccharide oxidases capable of oxidizing a variety of carbohydrates and possibly involved in the biotransformation of lignocellulosic compounds [64].

Concerning (hemi)cellulose bioconversion, genes encoding alpha-L-fucosidase (EC:3.2.1.51) (families GH29 and GH95 in CAZy) and alpha-L-arabinofuranosidases (E.C. 3.2.1.55) (GH51 and GH54) were abundant in RWS (T1) and also in TWS (T10). The alpha-L-arabinofuranosidases and alpha-L-fucosidases are the most important (hemi)cellulosic accessory enzymes that catalyze the hydrolysis of arabinans, arabinoxylans and alpha-l-fucosyl residues in agricultural waste [65-67]. In an anaerobic microbial community decomposing poplar wood chips, high levels of genes for these enzymes were found, especially in Bacteroidetes genomes [30]. In our microbial consortia, such genes may have come from *Sphingobacterium* members.

Endo-1.4-beta-xylanases (EC:3.2.1.8) (GH families 5, 8, 10, 11, 43) perform the endohydrolysis of (1 - 4)-beta-D-xylosidic linkages in xylans. These genes were also predicted to occur in both our consortia. Other genes involved in the deconstruction of xylan were also identified; for instance, the gene that encodes xylan-1,4-beta-xylosidase (EC:3.2.1.37) was enriched in T10 in RWS (Table 1). Beta-xylosidases are exotype glycosidases that hydrolyze short xylo-oligomers to single xylose units [68]. These enzymes were active in the secretomes of both microbial consortia, suggesting the expression of these bacterial genes.

The beta-galactosidases, which hydrolyze beta-galactosidic bonds between galactose and its organic functional group and can act on xyloglucans [69], were highly active in both consortia (Figure 6B). The beta-mannosidases (EC:3.2.1.25), involved in the hydrolysis of terminal, non-reducing beta-D-mannosyl residues in beta-D-mannosides [70], were lowly abundant in our consortia as compared to SS, but such activities were also detected in the secretome. The activities of these last two types of enzymes were higher in TWS1 than in RWS (Figure 6B), suggesting the raised availability of beta-D-galactose and beta-D-mannosyl residues in TWS, possibly released due to the torrefaction.

Conversely, mannan endo-1,4-beta-mannosidase (E.C: 3.2.1.78) (GH5, GH26, GH113 and AA10) related genes were enriched in RWS compared to SS. These enzymes are involved in the random hydrolysis of (1 - 4)-beta-D-mannosidic linkages in mannans, galactomannans and glucomannans. Cellobiohydrolases (endo- and exoglucanases) showed low activity in the secretome of TWS, suggesting the presence of high cellulose levels in the untreated compared with the torrefied wheat tissue. Several genes that encode beta-glucosidases were enriched in both consortia compared with SS, suggesting that the conversion of cellobiose to glucose is an important function in these consortia. Finally, cleavage and further metabolism of di-sugars was represented by several predicted enzymes. For example, alpha-L-rhamnosidase related genes were highly abundant in TWS at T10, compared to SS. These enzymes cleave terminal alpha-l-rhamnose from a large number of natural glycosides, and are relevant for application in citrus fruit juice and wine industries [71].

## Conclusion

In this study, the application of DNA-based high-throughput sequencing technology allowed the characterization of novel bacterial-fungal consortia growing on wheat straw. The data, in conformity with our previous work [29], indicate that mixed microbial consortia, encompassing specific biodegradative (mainly affiliated to *Klebsiella*, *Sphingobacterium*, *Flavobacterium*, *Acinetobacter*, *Penicillium* and *Acremonium*) and cheater types, are selected by the specific lignocellulose substrate. The approach allowed us to identify interesting yeasts, such as *Coniochaeta* and *Trichosporon*, that are possibly involved in plant biomass degradation and/or conversion of furanic compounds. Application of PICRUSt to predict the functional profile (using 16S rRNA sequences), in conjunction with the evaluation of enzymatic activities in the consortial secretomes, allowed the inference of genes/proteins that were presumptively involved in lignocellulose degradation (such as peroxidases, beta-mannases, beta-galactosidases, alpha-L-fucosidases, alpha-L-arabinofuranosidases and beta-glucosidases). Finally, assays of the degradation of other plant waste and quantification of initial and final products (for example, cello-oligosaccharides) might demonstrate the degradative potential that is needed for future biofuel production. A closer analysis of the metagenome and mobilome in our consortia will clarify the enzymatic profile and biotechnological potential present and can also shed light on the potential role of HGT in its evolution. A greater understanding of the ecological interactions between consortium members during plant biomass biodegradation is required for further progress in this area.

## Methods

### Lignocellulolytic microbial consortia construction

Soil samples (n = 10) were collected and mixed from a forest (top layer, 0 to 10 cm depth) in Groningen, The Netherlands (53.41 N; 6.90 E). Cell soil -suspensions were prepared by adding 10 g of fresh sampled soil to 250-mL flasks containing 10 g of sterile gravel in 90 mL of mineral salt medium (MSM). The flasks were shaken for 20 min at 250 rpm, and aliquots (250 μL) of soil suspension were added to triplicate Erlenmeyer flasks containing 25 mL of MSM with 1% lignocellulose substrate (0.25 g in 25 mL), amended with a trace element and vitamin solution. Two different substrates were thus obtained, to serve as carbon sources: *i*) “raw” wheat straw (RWS) and *ii*) heat-treated (torrefied) wheat straw (TWS). The flasks were incubated at 25°C with shaking at 100 rpm. Cultures were monitored at regular time intervals, and once the systems reached high cell density (log 7 to 8 cells/mL), aliquots (25 μL microbial suspension with fibrous material) were transferred to 25 mL of fresh medium. Finally, a sample of soil suspension and duplicate flask samples (selected based on reported DGGE analyses) at the final batches were taken from the RWS and TWS consortia after 1 (T1), 3 (T3) and 10 (T10) transfers (n = 13) and used for total DNA extractions and pyrosequencing as described below. Details of the experimental setup, substrate preparation, growth in sequential-batch cultures (cell counts and qPCR) and negative controls have been reported [29].

### Total DNA extraction and bacterial 16S rRNA/fungal ITS1 pyrosequencing

The DNA extraction from the cultures and from the soil suspension was performed with the Power Soil DNA extraction kit (MoBio® Laboratories Inc., Carlsbad, CA, USA) according to the manufacturer’s instructions. The bacterial 16S rRNA gene amplicons were generated using the universal primer set GM3F (5′-TAGAGTTTGATCMTGGC-3′) and 926R (5′-TCCGTCAATTCMTTTGAGTTT-3′) [72]. For fungal communities, specific primers ITS1F (5′-CTTGGTCATTTAGAGGAAGTAA-3′) and ITS4 (5′-TCCTCCGCTTATTGATATGC-3′) were used to amplify the ITS1, 5.8S rRNA and ITS2 regions of fungal rRNA [73]. The pyrosequencing reactions were performed with the new flow pattern B (software v2.8) and the FLX-Titanium chemistry (Roche/454 Life Sciences) at LGC Genomics (Berlin, Germany). Sequencing of 13 samples resulted in totals of 117,042 and 35,506 raw reads for bacterial 16S rRNA gene and fungal ITS1, respectively.

### Sequencing processing and statistical analysis

Pyrosequencing raw data were processed using the Quantitative Insights Into Microbial Ecology (QIIME) toolkit [74]. The sequences were quality trimmed using the following parameters: quality score of >25, sequence length of 300 to 900 bp (for 16S rRNA) and 100 to 900 bp (for ITS1), maximum homopolymer of 6, 0 maximum ambiguous bases and 0 mismatched bases in the primer. In order to select for the same region of each gene, we retrieved sequences with primers GM3F (for the bacterial 16S rRNA) and ITS1F (for fungal ITS1). We identified bacterial and fungal players by grouping highly similar sequences into OTUs (at 97% of nucleotide identity) using UCLUST [75] followed by selection of representative sequences. Subsequently, chimeric sequences were detected using ChimeraSlayer [76] and deleted. Additionally, clusters consisting only of singleton sequences were removed in order to avoid sequencing errors. Analyses of community composition, as well as richness and diversity estimators, were carried out at a depth of 1,400 bacterial and 550 fungal rarefied sequences per sample, to eliminate the effect of sampling effort. QIIME was also used to generate alpha- and beta-diversity metrics, including OTU richness, CRE, SWI, PD and UniFrac distances. Taxonomic classifications at the phylum and order level of each OTU were done using RDP classifier and BLAST algorithms against the Greengenes (16S rRNA), UNITE and GenBank (ITS1) databases. The assignment of each OTU on the genus level was based on the best BLASTn hit against the GenBank database (Additional file 6). Abundant OTUs, more than 10 and 5 sequences per OTU for the 16S rRNA and ITS1 data respectively, were selected to construct the PCA using Canoco software v4.52 (Wageningen, The Netherlands). The 16S rRNA and ITS1sequences were deposited in GenBank with SRA accession numbers [SRP039495].

### Detection of abundant OTUs as bacterial strains

Isolation of bacterial strains along the experiment and the determination of their taxonomic identification and (hemi)cellulolytic activity in agar plates (with CMC and xylan from birchwood) were previously reported [29]. Partial 16S rRNA gene sequences of these strains were obtained using the same forward primer as used for the 16S rRNA pyrosequencing. To detect which OTUs were possibly recovered as bacterial strains, we constructed a phylogenetic tree using the sequences of the 15 most abundant bacterial OTUs (representing over 72% and 88% of the consortia in RWS and TWS, respectively) in addition to 20 sequences retrieved from the bacterial strains. Sequences were aligned using the ClustalW software, and the phylogenetic analyses (*p*-distance) were conducted with MEGA v5.1 using the Neighbour-Joining method [77]. The evolutionary distances were computed using the Kimura-2 parameter method and are in the units of the number of base substitutions per site (note scale bar - Figure 5). The branches were tested with bootstrap analyses (1,000 replications). Furthermore, (hemi)cellulolytic activity was linked to the OTUs based on the similarity and clustering with the bacterial strains.

### Reconstructing the bacterial metagenomes with PICRUSt software

The bacterial metagenomes were reconstructed using the PICRUSt software [27]. A PICRUSt-compatible OTU table was constructed in QIIME (at 97% of nucleotide identity) using the newest available reference closed-reference OTU collection in the Greengenes database [78]. In order to normalize the data, we used 1,000 rarefied sequences of bacterial 16S rRNA per sample as an input. Subsequently, the normalization by 16S rRNA copies number per OTU was performed with the *normalize_by_copy_number.py* script and IMG database information. The metagenome inference was done using the *predict_metagenomes.py* script with the normalized OTU table as an input. We analyzed the average number of annotated genes in each sample and selected the top 40 known genes related with the bioconversion of lignocellulose. PICRUSt also calculated the NSTI, a measure of prediction uncertainty presented here in a comparative way along the sequential batches in both consortia datasets.

### Quantification of specific enzymatic activities related to the (hemi)cellulose bioconversion

In order to evaluate the metabolic potential in the degradation of (hemi)cellulose and the expression of selected genes identified by the PICRUSt prediction, we quantified specific enzymatic activities in samples of 2 mL from the enriched cultures after final batch (T10), when the communities are stable. Microbial cells plus wheat substrate were harvested by centrifugation for 3 min at 12,000 rpm, the supernatant (secretome) was recovered and tested for enzymatic activity using MUF-beta-D-xylopyranoside, MUF-beta-D-mannopyranoside, MUF-beta-D-galactopyranoside, MUF-beta-D-cellobioside and MUF-beta-D-glucopyranoside as substrates. The reaction mixture consisted of 10 μl of MUF-substrate (10 mM in dimethyl sulfoxide), 15 μL of Mcllvaine buffer (pH 6.8) and 25 μL of each supernatant. The mixture was incubated at 27°C for 45 min in the dark, and the reaction was stopped by adding 150 μL of 0.2 M glycine-NaOH buffer (pH 10.4). Fluorescence was measured at an excitation of 365 nm and emission of 445 nm. We also evaluated the fluorescence without the MUF-substrate as a negative control. Enzyme activities were determined from the fluorescence units using a standard calibration curve and expressed as rates of MUF production (nM MUF per min at 27°C, pH 6.8).

## Abbreviations

AA: auxiliary activities; CAZy: Carbohydrate-Active EnZymes database; CMC: carboxymethyl cellulose; CRE: Chao richness estimator; DGGE: denaturing gradient gel electrophoresis; GH: glycosyl hydrolases; HGT: horizontal gene transfer; MSM: mineral salt medium; MUF: 4-methylumbelliferyl; NSTI: Nearest Sequenced Taxon Index; OTU: operational taxonomic unit; PCA: principal components analysis; PD: Faith’s phylogenetic diversity; PICRUSt: Phylogenetic Investigation of Communities by Reconstruction of Unobserved States; QIIME: Quantitative Insights Into Microbial Ecology; qPCR: quantitative polymerase chain reaction; RA: relative abundance; RWS: untreated wheat straw; SIP: stable isotope probing; SS: soil inoculum; SWI: Shannon-Wiener index; TWS: heat-treated wheat straw.

## Competing interests

The authors declare that they have no competing interests.

## Authors’ contributions

DJJ designed and constructed the experiments, participated in the sequence analyses and drafted the manuscript. FD-A carried out the bioinformatic and statistical analyses. JDvE conceived the study and participated in its design and coordination and helped to draft the manuscript. All authors read and approved the final manuscript.

## Supplementary Material

Additional file 1 **Rarefaction curves in the soil inoculum (SS) and in enriched cultures (RWS and TWS) along the sequential batches.** Rarefaction curves of (A) bacterial 16S rRNA and (B) ITS1 pyrosequencing. OTUs were generated at 97% of nucleotide identity. Click here for file

Additional file 2 **Relative abundance (bacteria) in the soil inoculum (SS) and in enriched cultures (RWS and TWS) along the sequential batches.** Relative abundance (%) of the most abundant bacterial (A) phylum and (B) orders based on 1,400 (16S rRNA) rarefied sequences. Click here for file

Additional file 3 **Relative abundance (fungi) in the soil inoculum (SS) and in enriched cultures (RWS and TWS) along the sequential batches.** Relative abundance (%) of most the abundant fungal (A) phylum and (B) orders based on 550 (ITS1) rarefied sequences. Click here for file

Additional file 4 Unweighted UniFrac three-dimensional continuous matrix of bacterial 16S rRNA and ITS1 pyrosequencing data from soil inoculum (SS), RWS and TWS consortia in different sequential batches. Click here for file

Additional file 5 **Number of OTUs per taxon in the soil inoculum (SS) and in enriched cultures (RWS and TWS) along the sequential batches.** Number of OTUs per taxon of the most abundant (A, B) bacterial orders and (C, D) fungal phylum based on 1,400 (16S rRNA) and 550 (ITS1) rarefied sequences. Click here for file

Additional file 6 **OTU table with the taxonomic affiliation.** OTU table with the taxonomic affiliation of each OTU (bacteria and fungi) based on BLASTn against GenBank database. The table shows the number of sequences per OTU in enriched cultures (RWS and TWS) and along the sequential batches. Click here for file
