# Supplementary figures and images for: Metataxonomic profiling and prediction of functional behaviour of wheat straw degrading microbial consortia
Source: Biotechnol Biofuels. 2014 Jun 12;7:92. doi: 10.1186/1754-6834-7-92 (PMC4064818; doi:10.1186/1754-6834-7-92)

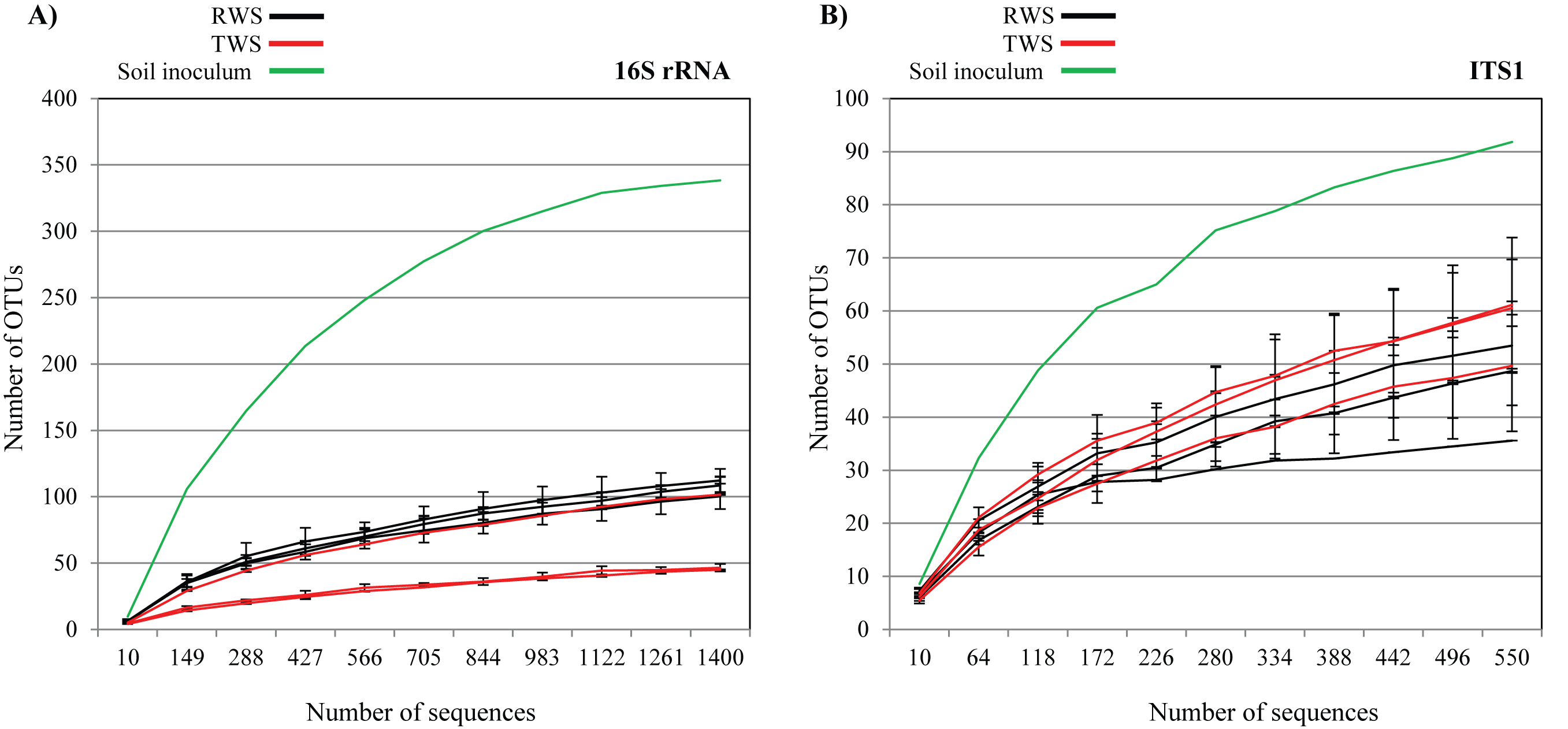

Supplement: Additional file 1 — Rarefaction curves in the soil inoculum (SS) and in enriched cultures (RWS and TWS) along the sequential batches. Rarefaction curves of (A) bacterial 16S rRNA and (B) ITS1 pyrosequencing. OTUs were generated at 97% of nucleotide identity. [file 1754-6834-7-92-S1.tiff]

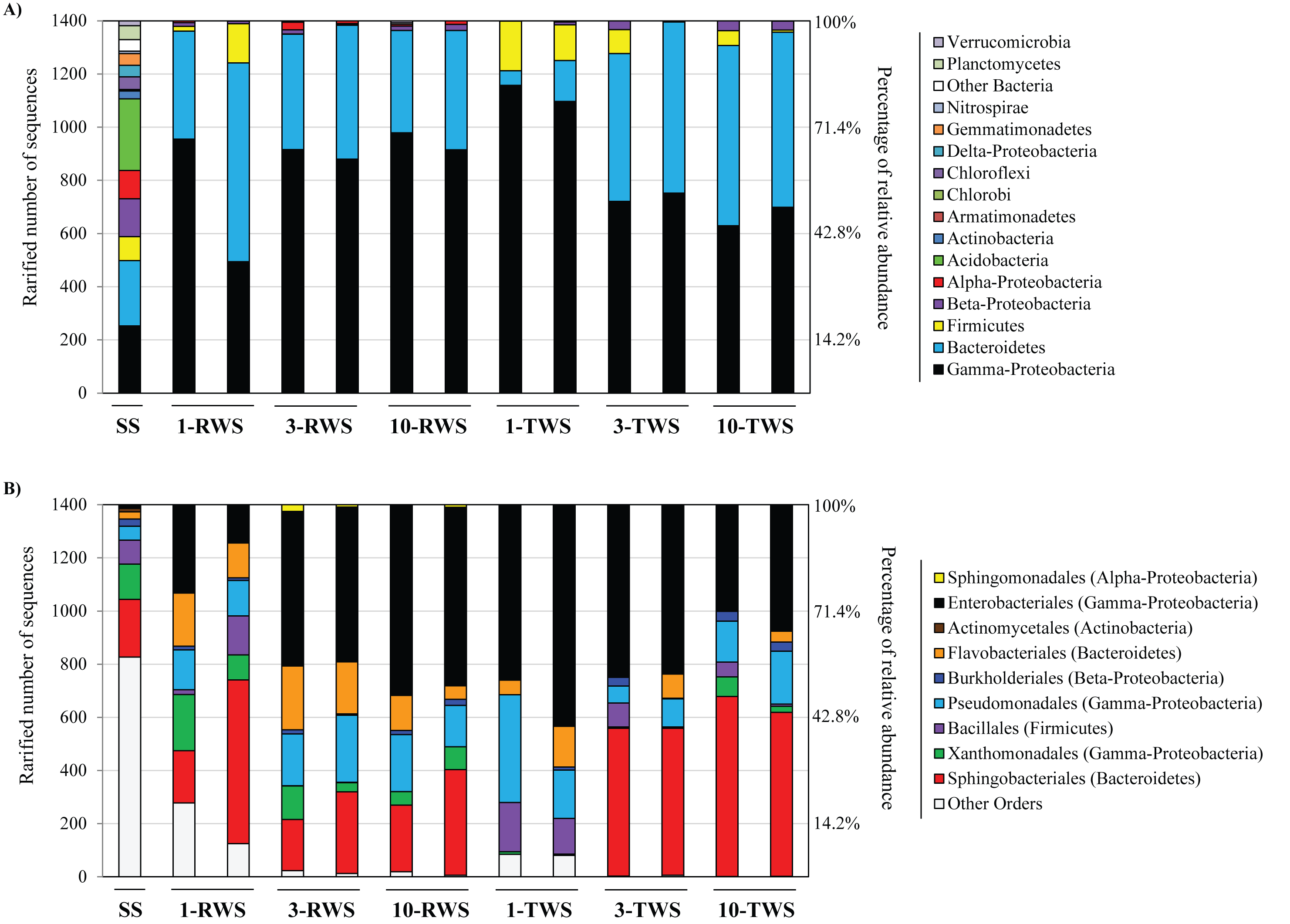

Supplement: Additional file 2 — Relative abundance (bacteria) in the soil inoculum (SS) and in enriched cultures (RWS and TWS) along the sequential batches. Relative abundance (%) of the most abundant bacterial (A) phylum and (B) orders based on 1,400 (16S rRNA) rarefied sequences. [file 1754-6834-7-92-S2.tiff]

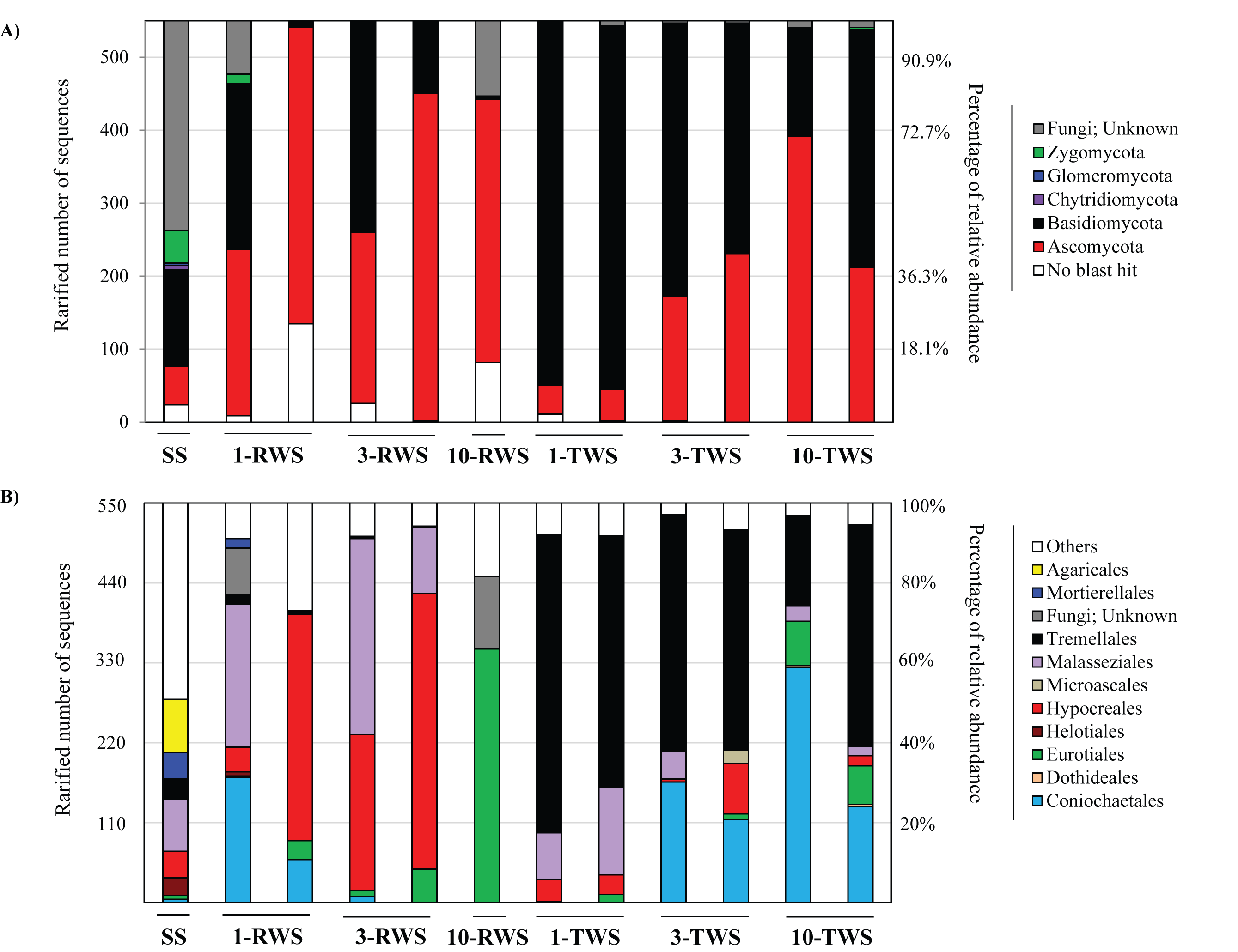

Supplement: Additional file 3 — Relative abundance (fungi) in the soil inoculum (SS) and in enriched cultures (RWS and TWS) along the sequential batches. Relative abundance (%) of most the abundant fungal (A) phylum and (B) orders based on 550 (ITS1) rarefied sequences. [file 1754-6834-7-92-S3.tiff]

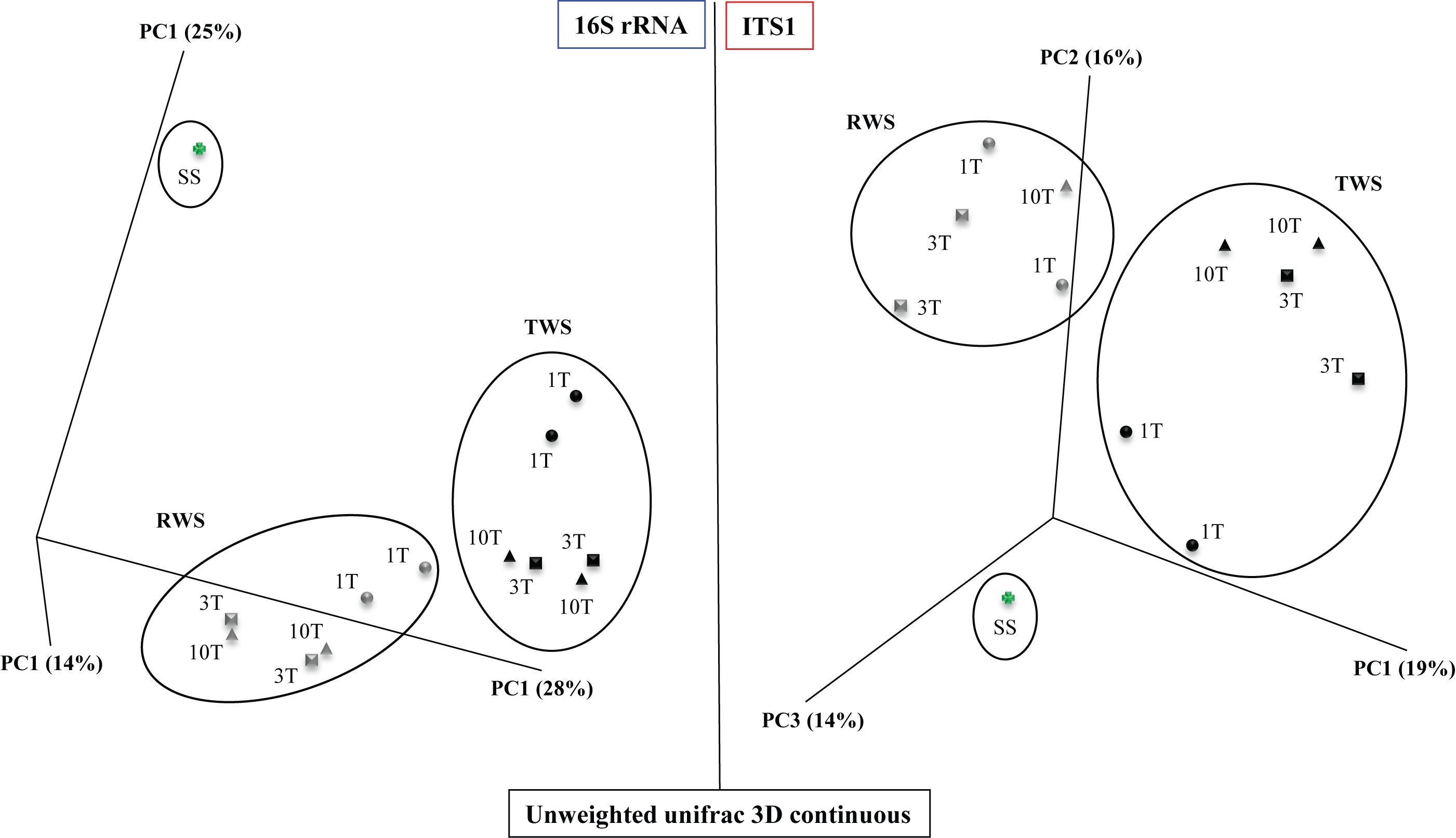

Supplement: Additional file 4 — Unweighted UniFrac three-dimensional continuous matrix of bacterial 16S rRNA and ITS1 pyrosequencing data from soil inoculum (SS), RWS and TWS consortia in different sequential batches. [file 1754-6834-7-92-S4.tiff]

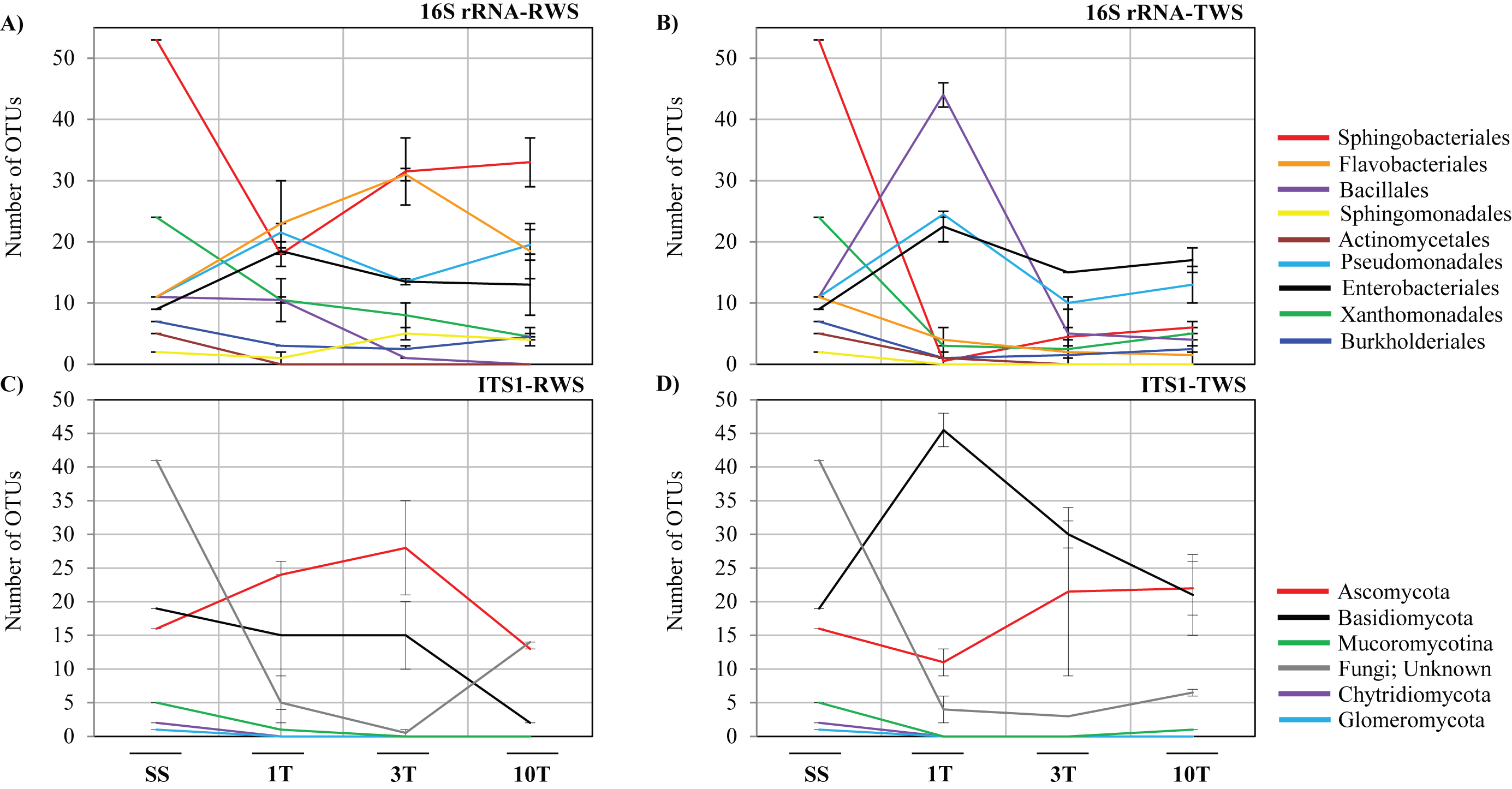

Supplement: Additional file 5 — Number of OTUs per taxon in the soil inoculum (SS) and in enriched cultures (RWS and TWS) along the sequential batches. Number of OTUs per taxon of the most abundant (A, B) bacterial orders and (C, D) fungal phylum based on 1,400 (16S rRNA) and 550 (ITS1) rarefied sequences. [file 1754-6834-7-92-S5.tiff]
